# Free-Form Dance as an Alternative Interaction for Adult Grandchildren and Their Grandparents

**DOI:** 10.3389/fpsyg.2020.00542

**Published:** 2020-04-17

**Authors:** Einat Shuper Engelhard

**Affiliations:** ^1^Graduate School of Creative Art Therapies, Faculty of Humanities & Social Sciences, Kibbutzim College of Education, Tel Aviv, Israel; ^2^Graduate School of Creative Art Therapies, Faculty of Social Welfare & Health Sciences, Emili Sagol Creative Arts Therapies Research Center, University of Haifa, Haifa, Israel

**Keywords:** adult grandchildren, alternative interaction, dance, dance/movement therapy, elderly, free-form dance, grandparent

## Abstract

Shared leisure activities fulfill a central role in strengthening the relationship with adult grandchildren and provide a vehicle for transmission of values. Thus, it is likely that joint activity alongside support and focused guidance in communication between adult grandchildren and grandparents will help strengthen their relationship. Based on theory and research on dance/movement therapy in old age, and on development processes in the family, the aim of the present study is to discover the significance of free-form dance jointly engaged in by adult grandchildren with their grandparents, for each of the generations. Using action research, 16 dance-movement therapists and their grandmothers participated in three dance meetings in their grandmothers’ homes. Based on filmed videos of the sessions, personal diaries, and semi-structured interviews, it was found that among the granddaughters, the meeting aroused concerns about their lack of skill to create a meaningful meeting and to protect their grandmothers throughout the meeting. It was also found that regular free-form dance meetings in which the granddaughter mirrored her grandmother’s movements while suggesting expansions to the movements, encouraging eye contact, touch, and playfulness, and empowering her ability, while also granting legitimacy to rest, created a change in the grandmother’s state of mind: positive memories and feelings appeared, as did uplifted spirits. For the granddaughters, the meeting altered their perspective on old age and provided a space for processes of parting. The implications of identifying assistive components of joint dancing for creating an intervention model for adult grandchildren’s support of the elderly in the community are extensively discussed.

## Introduction

The over-age-65 population has consistently grown and is likely to double and constitute 22% of the population by 2050 (World Health Organization, 2018). Thus, the search for targeted interventions relevant to the full range of physical, mental, and social aspects of the older person is needed (Mcneill and Anstey, 2016). These are precisely the areas influenced by dance in old age: dance significantly improves muscle strength, endurance, balance, regulation, and motor control; prevents anxiety and depression; and is of help in dementia (Hwang and Braun, 2015). The aim of the present study is to examine the significance of joint free-form dancing in meetings between grandparents (GPs) and their adult grandchildren (GCs) for each of the parties.

### Dancing in Old Age and the Uniqueness of Dance/Movement Therapy

The impact of dance on the elderly cuts across cultures. In a study conducted in a community center in South America, it was found that dance had a positive impact not only on physical aspects of old age but also on self-perception, psychological well-being, social integration (Vadineia et al., 2016), self-esteem, and mood (Silva et al., 2016). In a study conducted in the United Kingdom that examined the effect of social dancing on an older adult population, it was found that dance was a means of self-discovery and a sense of worth (Cooper and Thomas, 2002). For older people with dementia, dance expands limited expression and social communication and enhances quality of life (Lelièvre et al., 2015; Pereira-Stubbs, 2018). Weekly dance sessions provide a communal experience, mutual support, a sense of worth, respect, enjoyment, and autonomy (Schneider, 2018).

In parallel to the positive impact of dance, in the above-age-60 population, there are physical and medical limitations as well as issues of available time, funds, and cultural perceptions that lead to a decline in physical activity (Chong et al., 2012). Dance/movement therapy (DMT) bridges the gap between the advantages of dance and the difficulties in performing physical activity and offers a combination of expressive movement and creativity (which does not require any physical abilities or prior knowledge of movement), along with emotional discourse aroused in consequence of the movement (European Association Dance Movement Therapy, 2018). Extensive theoretical (Bräuninger, 2014; De Tord and Bräuninger, 2015) and clinical (Guzmán et al., 2016; Low et al., 2016) bases show that the unique framework of this treatment provides physical activity adapted to the physical, emotional, and social needs and limitations of the older person.

In DMT, the therapist invites the participant to engage in free-form movement, whose characteristics the therapist mirrors. This, along with the therapist’s familiarity with the significance of prominent motifs in each individual’s personal movement profile, enable the therapist to promote cognitive and social facets and verbal and non-verbal expressions in a safe and supportive environment (Shuper Engelhard, 2017). The therapist’s sensitive attunement to the individual’s spontaneous movement serves to encourage its expansion and encounter its resources; as such, it represents a source for an experience of renewal and development of mental resilience in old age (Capello, 2018). Moreover, in research with various populations, it was found that DMT makes a significant contribution to the reduction of anxiety and depression (Koch et al., 2013) prevalent in old age (World Health Organization, 2018).

In studies conducted in senior homes, it was found that DMT with older people with dementia contributes to cognitive, motor, and social functioning (Hokkanen et al., 2008; Duignan et al., 2009; Karkou and Meekums, 2017). For those moving into sheltered housing, DMT contributes to an increased sense of belonging, emotional well-being, and social involvement (Kluge et al., 2012). Similarly, several studies found that DMT interventions contribute positively to older adults with mental health disorders (Jiménez et al., 2019), whereas DMT’s contribution to the well-being of older adults living in the community (not in homes for the elderly) has not been examined to date. It is possible that this is related to the lack of DMT training in community settings and to the difficulty in recruiting participants for an intervention that requires leaving the house, and due to the expense of therapy. All these necessitate examination of methods for transferring the focus of treatment to the home, to expand the circle of the older person’s support within the family framework and to provide the family with tools to care for the elderly person.

### Relationships Between Adult Grandchildren and Their Grandparents

Only a few research studies have highlighted the unique dynamic of the relationship between adult GCs and their GPs (Scharf, 2016; Silverstein, 2019). Adult GCs are not dependent upon their parents to make a decision as to the frequency and quality of their relationship with their GP. They describe their relationship with their GP as one of mutual support and similar to friendship (Kemp, 2005). Close relationships between GPs and adult GCs are tied to better mental health in the two generations, including lower levels of pressure, depression, and loneliness (Even-Zohar and Tzurit Garby, 2016). In addition, the GC’s involvement in the GP’s life relieves and helps the family’s “main caregiver” and contributes to the older person’s quality of life (Sheehan and Petrovic, 2008).

In research on the quality of relations throughout the years, a single study found that there was a decline in the relationship with the GP during adolescence (Geurts et al., 2009); in contrast, other studies found that the quality of the relationship is preserved throughout the life span (Hakoyama and MaloneBeach, 2013). A close relationship during childhood increases the likelihood of a close relationship in adulthood (Geurts et al., 2012) as well as provision of assistance to the GP (Silverstein and Xu, 2016). Moreover, when the relationship in childhood is distant, the GP does not expect a close relationship during young adulthood (Pusateri et al., 2016). Thus, it can be assumed that a less-close relationship with the GP during childhood will need support and guidance in order for change to take place in the relationship during the life span.

Other studies have shown that the level of comfort felt by adult GCs when listening to their GP’s painful self-disclosures was linked to closer relationships (Fowler and Soliz, 2010). Accordingly, open communication with the GP increases GCs’ sense of familial belonging (Soliz and Harwood, 2006) and is related to more positive attitudes towards the elderly (Tam et al., 2006) and to greater closeness (Scherrer, 2016).

Generational differences in the perception of values and worldviews is another variable that can create communication difficulties between the generations. At the same time, it was found that shared leisure activities fulfill a central role in strengthening the relationship with adult GCs and provide a vehicle for transmission of values (Hebblethwaite, 2016; Silverstein and Bengtson, 2018). Thus, it is likely that joint activity alongside support and focused guidance in communication between adult GCs and GPs will help strengthen their relationship.

One study on adolescent GCs and dementia shows that they are interested in maintaining a close physical and emotional relationship with the older adult while not knowing how to do so (Celdrán et al., 2014). A recent study in which students participated (Miron et al., 2019) examined the nature of the participants’ concern and their coping strategies during face-to-face interactions with their GPs who were diagnosed with dementia. The GCs mentioned concerns regarding their lack of skill in reinforcing and preserving relations, not knowing what to say or how to behave, paucity of skills needed for conducting themselves in the situation, and few strategies for regulating emotion, alongside difficulty in interacting with a constantly changing other.

### Goals of the Study

Despite the importance of intergenerational relations, the contribution of guided, structured activity to encounters between the older adult and his or her adult GCs has not been studied to date. The current study is the first of its kind, and its aim is to examine how accepted DMT techniques can serve the generation of adult GCs in the attempt to provide physical, cognitive, emotional, and spiritual support to the older person. As the literature suggests, as the aging process progresses, intervention to support alternative ways of communicating with GPs is needed (Miron et al., 2019) and will provide the ground for shared activities (Hyden, 2014; Majlesi and Ekström, 2016). DMT provides tools for an intimate encounter that facilitates self-disclosure and emotional empowerment (Shuper Engelhard, 2017, 2019b). As mentioned, to date, the contribution of a DMT-mediated intervention with older persons in the community has not been examined, and neither has the contribution of tools from the DMT field to the interaction of GCs with their GPs. In addition, the connection between adult GCs was examined only with respect to a sample of adolescents and students (young adults). This study pertains, for the first time, to GCs who are mature adults.

The assumption underlying the present research is that joint dancing of an adult GC with his or her GP will contribute to improving the relationship and to familiarity between them, and will improve the older person’s physical, social, and mental status. Due to the nascent nature of work in this field, the goal of the current study is to learn about the emotional and mental contribution of dyadic experiencing of free-form dance by granddaughters (GDs) who are dance/movement therapists with their grandmothers (GMs). The intention is for this to serve as a foundation for developing a community intervention model that will provide support to adult GCs in strengthening the relationship with the generation of GPs. The current study focuses on the viewpoints of the GM and the GD during their joint meeting, so as to provide an expansive picture of the dynamics of the relationship (Celdrán et al., 2014) and to answer the following questions: What concerns do the GDs harbor with respect to the dance encounter with their GMs? In what way does dance contribute to relations between GDs and their GMs and to their communication? Which elements of dance assist the older adult, and with which areas?

## Materials and Methods

### Research Participants

Sixteen dance/movement therapists between the ages of 30 and 45 (*M* = 36, SD = 2.4) whose GMs ranged in age from 80 to 95 (*M* = 88, SD = 0.32) responded to a call issued on social media for participation in a study. The call for participants was made to men and women alike, and it is probable that the response of women only was related to the DMT profession’s majority of women. The participants came from diverse backgrounds with respect to religion, origin, and culture. Among the GMs, 4 used walkers, and 14 reported on a decline in memory. Two of the GMs had severe short-term memory impairment. The GDs typically met with their GMs once in 3 weeks. All lived an average of 1 h traveling distance from their GM. One-third of the GMs regularly engaged in physical activity for an average of 2 h weekly. In recent years, all had been suffering from health problems and decreasing independence. All the GDs reported on the family’s feeling of helplessness with respect to the deterioration in the GM’s health.

This research was approved by the ethics committee at the academic institution by which the researcher is employed.

### Data Collection

Sixteen dance/movement therapists and their GMs participated in three dance meetings within the framework of participatory action research (action research is an approach in which the action researcher and a client collaborate in the diagnosis of the problem and in the development of a solution based on the diagnosis; Bryman and Bell, 2011), which took place in the GM’s home where just the two were present. Due to the germinal nature of the research in this field, the participants’ professional background helped identify key topics in the field (Keirnan, 1999). With the aim of facilitating continuity and familiarity with the meaning of an ongoing process, the meetings took place at 1-week intervals. All the meetings were identical in structure. The GDs were instructed to clear space in an area suitable for movement in their GM’s home (move aside a table in the living room, etc.), to prepare the camera and tripod to film the meeting, and to invite their GM to move along with the music chosen by the GM. To start off, the GDs were asked to suggest that their GMs lead the dance, after which the GD would lead it. In accordance with key DMT techniques, the GDs were asked to be attentive to their GM’s movement and move as a reflection, to mirror it, and to propose expansion to it while being sensitive to the individual nuances of the movement (such as the intensity of muscle use, rhythm, space) (Koehne et al., 2016). The invitation is to free-form dancing, which is not structured in advance, can be performed while sitting or lying down, and does not require any previous knowledge or skills. Beyond these instructions, the GDs were asked to be flexible and sensitive to their GM’s needs in each meeting and to respond as needed. The duration of the joint dance was between 10 and 15 min (similar to the structure of the mirror game, a frequent exercise used in DMT; for more information, see Feniger-Schaal et al., 2018).

The GDs kept a diary all through the sessions. They were asked to detail why they chose to participate in the study and what their feelings were about the dance meetings with their GMs. Writing a diary facilitated free-from writing about thoughts, feeling, and memories (Aldwin and Revenson, 1987) and helped define the GD’s personal experience of the interaction with her GM. At each meeting, following their joint dance, the GDs were asked to record in their diaries what they felt about the movement, the relationship, their GM, and old age, and how they perceived their GM’s experience with reference to all these. They were also asked to describe what characterized their own and their GM’s movement suggestions and, in their opinion, the reason these characteristics appeared in the movement. Afterwards, they interviewed their GMs using a semi-structured interview guide given to them in advance and in which they asked their GM to describe the significance of the dance to her and to describe emotions, feelings, and memories that arose during the dance.

### Data Analysis

The stated and implicit significance of structured, free-form dance meetings of GMs and adult GDs was examined using interpretive phenomenological analysis (IPA) (Smith, 1996) of the video transcriptions, the personal diaries, and the interviews. This method has been used in the past in research on adults with dementia (Clare, 2003; Miron et al., 2019) and is unique in its focus on the subjective experience of the research subject along with its recognition of the role played by the investigator’s interpretation (Quinn and Clare, 2008). Data analysis was performed by the study author, who is an expert in the field of gerontology and DMT, and by two additional experts in action research and family relations.

During the first stage, all the diaries, video transcriptions, and interviews were analyzed by identifying relevant topics and dividing topics into clusters. In the second stage, a list of themes was compiled. In the third stage, all the materials were reread so as to ensure all topics were identified; this resulted in a complete list of relevant themes for each topic. In the fourth stage, the data and the topics were checked by an additional researcher experienced in use of the IPA method and by experts in dementia and DMT. At this stage, the GDs were invited to a focus group where there was an atmosphere of acceptance and lack of judgment (Krueger and Casey, 2015), in which the themes uncovered were conveyed to them and they were asked to share their feelings, emotions, and thoughts about them with the aim of gaining a more precise familiarity with the less conscious experiences (Henry and Fetters, 2012).

## Results

All the GDs expressed concern with respect to the responsibility for creating an encounter that would be significant and for safeguarding and protecting their GMs during the meeting. Each of these is a separate, legitimate concern, though for all the pairs, the first one dissipated towards the third meeting, while the concern about safeguarding their GM persisted for some even into the third and final session (*n* = 3). At the same time, they stated that the sessions painted old age in a new light and made possible a valuable space for eventual parting. The GDs and their GMs felt dance changed the “state of mind” of old age: it empowered positive perception and emotions and led to an uplifted spirit. Based on all the GM–GD pairs, it emerged that these implications depend on the framework for the meetings and on the nature of the instruction given by the GD (see Tables 1–3).

**TABLE 1:** Granddaughters’ concerns and dilemmas with respect to the meetings.

**(I) Will I be able to create a meaningful meeting?**	**(II) Will I be able to safeguard and protect?**
(1) Concern over embarrassment and shame	(1) Concern about the grandmother‘s tendency to want to please
(2) Concern over lack of significance	(2) Concern regarding the aging body
(3) Concern about lack of desire and energy	(3) Concern regarding overwhelming and frightening emotions

**TABLE 2:** Significance of free-form dance in meetings of adult granddaughters with their grandmothers.

**For the granddaughter**		**For the grandmother**
**(I) Puts old age in a new light**	**(II) Provides space for eventual parting**	**(III) Changes the state of mind**
(1) The body is surprising in its youth	(1) The need to create shared memories	(1) Empowers a positive perception
(2) Decreases the generation gap	(2) The need to be thankful and to give back	(2) Empowers gestures of love
(3) Expands the roles in the relationship	(3) The need to create closeness/intimacy	(3) A means by which to feel loved
(4) Reveals strength of character	(4) Permission to let go, ask, and feel	(4) Leads to uplifted spirits

**TABLE 3:** Unique components for a successful meeting.

**(I) Meeting framework**	**(II) Nature of granddaughter’s instruction**
(1) Movement accompanied by closely related partner	(1) Breathing, touch, eye contact, playfulness
(2) Regularity and repetition	(2) Joining in and gradually expanding the movement
(3) Focus on connection and enjoyment, not on form of the movement	(3) Giving legitimacy to rest
(4) Spontaneous, not structured, movement	(4) Encouraging and appreciating grandmother’s ability

### Granddaughters’ Concerns and Dilemmas With Respect to the Meeting

#### The Concern That I Will Not Be Able to Create a Meaningful Meeting

The majority of GDs (*N* = 12) shared that despite their close relationship with their GM they had never tried to dance with her or even saw her dancing. Thinking about the meeting aroused feelings of embarrassment and worry regarding their ability to successfully engage in it: “Movement with Grandma is new, strange and unfamiliar to me. Our relationship is one of talking. I worry that we won’t know what to do, that I’ll be embarrassed to suggest that she move, that it’ll be odd for her or that she’ll want to stop in the middle.” And, “I’m anxious about the embarrassment, that something won’t flow and I won’t know how to change course so that it’ll be easier; I wonder if my grandmother will lead the movement and how she’ll do it. I hope we’ll feel at ease.”

All of the GDs expressed concern regarding their needs in contrast to those of their GMs and worry that the meeting may lack meaning. This was especially prominent when the GM danced without making eye contact with the GD. In such instances, the GDs felt “confused,” “helpless,” and “disappointed.” “It seems that Grandma is in her own world when she dances; I had the feeling that it made no difference if I was there or if someone else was.” And, “It was hard for me that Grandma didn’t make eye contact with me… we dance together but I feel that she doesn’t need me.” These situations aroused a dilemma among all the GDs about whether and how to encourage a more intimate connection: “Most of the time I felt that Grandma didn’t need me, I felt I was trying hard to capture her gaze, she should be dancing with me. I was surprised and delighted with Grandma’s dedication to the dance. But I wondered whether and to what degree to mediate my need for her gaze.” Another concern (*N* = 6) was regarding the right course of action in situations where the GM showed a lack of desire and energy: “At our meeting, my grandmother didn’t feel well and didn’t want to get out of bed at all. I wondered whether it was right for me to put pressure on her.”

#### Concern That I Would Not Be Able to Safeguard and Protect Her

All the GDs expressed concern that in their wish to please their GD, their GM would not say the movement was hard for her: “When Grandma became tired, I found myself debating whether to continue or stop, I didn’t rely on her to stop.” And, “I fear and worry. She’s in pain. She’s tired and she continues to move and doesn’t want to stop.” Most of the GDs described the physical tension in their own bodies during the meeting, resulting from their worry for their GM’s welfare: “I noticed that when she moves backwards my senses are heightened and I worry, I was afraid.” With respect to the eventual separation old age brings with it: “Something in these meetings increased thoughts about the day we will part. It brought up a very great fear and sadness.”

Similarly, all the GDs expressed concern about the nature and intensity of the emotions that would be brought to the fore after the movement. Fear that the movement would bring up dormant memories that would harm their GM’s health: “I want to be careful not to raise demons with which she won’t be able to cope and I won’t be able to help her.” And, “Despite our closeness she never told me details about her past, she almost never spoke of her emotions. Suddenly, the movement brought up many memories and I was afraid for her wellbeing.”

### Significance of Adult Granddaughters’ Free-Form Dancing With Their Grandmothers

#### Old Age in a New Light

All the GDs felt that through the dance, their GMs’ bodies surprised them with their youth: “The intensity of the movement together with her facial expressions and hand movements really surprised me. Suddenly she moved with her pelvis and seemed free.” Through the dance, the GDs observed their GMs “beyond the aging body.” They discerned playfulness, sexuality, and amusement, which, until now, were not part of the image they had of their GMs: “When I look at her moving, old age is less frightening.” And, “It was moving and surprising to see how beautifully and freely she dances. I was given the opportunity to see her as a woman, as a person, not only as a grandmother.” All the GDs were surprised by the energy of their GM’s body: “I was surprised that when I jumped and moved quickly and Grandma mirrored my movement at my rhythm and with the same energy; I noticed at each meeting that her animation and vitality surprised me and her range of rhythms were as wide as mine.” And, “Her lightness and range of movement surprised me. This is how I remember her from when I was a girl.”

All of the GDs related to how the dance bridges the generation gap. The GDs related to the difference between conversation and movement: “I usually talk to her about things that are going well for me and less about difficulties; when I did share I felt the gap between us in how we look at things today versus in her time. Dance differs from talk because it’s something we share and we both enjoy.” And, “In the dance, I felt our age gap decrease. When we talk, the difference between her world and mine is obvious, dealing with health, doctors, and Grandma doesn’t always remember from one conversation to the next; in dance, our ideas are equal. I learn from her and she observes me and mirrors my movements, it’s another kind of encounter. I felt joy, closeness, love and great appreciation for her.”

Dance expands the roles in the relationship. It creates a meeting in which there is enjoyment, spirituality, and less care: “I’m very close with Grandma and lately I accompanied her to checkups. Suddenly, dancing with Grandma created something new, closer, in which there’s laughter and excitement.” And, “Today, as we began, I felt closer to her. Something in our relationship changed a little. Now I’m not only her youngest granddaughter but someone with the ability to help. Even when we sat in the yard, I noticed that we weren’t in our regular places, we switched.” The nature of the encounter enabled change: “My interest in her, being attentive to her rhythm, her needs, her pleasure, were significant for the both of us. A role reversal occurred which created uniqueness and closeness.” And, “I felt I was in the role of the adult, the instructor, the protector, the ‘professional.’ This focus was significant for us both.”

Dance exposed all the GDs to their GMs’ strength of character: “Owing to the meeting, I was able to see another side of my grandmother. I saw how much she fights, how persistent she is.” And, “My grandmother enjoyed moving very much, she gave it her all. Despite it being hard for her to stand, she exerted herself and moved in her chair, powerfully, clearly, with a lot of attention to herself and her needs.” Much of the energy was related to the connection with the GD: “It was pleasant and surprising when Grandma bent down. She exerted herself, didn’t hold on to her cane or to a pillar. She felt secure in using me as mental support.” And, “It feels strange to me to see her imitate my movements, secure, willing to try.”

#### The Dance Provides a Space for Eventual Parting

All the GDs spoke of the need to create shared memories: “To spend as much time with Grandma as possible. For her, or even more, for me.” After the meetings, the GDs were full of gratitude. Statements such as the following were repeated: “Thank you for making it possible for me to participate”; “This was a one-time opportunity that won’t recur, I wouldn’t have experienced it otherwise. It will remain with me forever”; “Thank you for this huge gift that was given to me”; and “This was an opportunity to give Grandma more moments in which her body feels different and for me, to be close to her in a new way.”

All the GDs expressed the hope to engage in a unique experience with their GMs: “This is different than the usual experience because it has a purpose and is planned. It’s just the two of us. Doing something together. I haven’t yet experienced something like it. I’ve never danced with her.” And, “I’m not sure I’ve ever seen her dance. She is a happy, good and pretty woman, brave and strong. And alone, so alone.” The sense that this is an “opportunity for an ‘other’ and ‘different’ type of meeting,” which will facilitate greater familiarity, came up again and again: “I’m especially curious whether there’ll be spontaneity, dependence and contact; my grandmother is independent, strong. I wonder what more I’ll learn about her through movement.” All the GDs expressed a longing for new intimacy in the relationship that the meetings can bring about: “I know that this will lead to something new in the relationship with Grandma.” And, “To move together with Grandma is to return to childhood, I remember that we used to dance together when I was a child and apparently it stopped when I got older.” The same experience is mentioned by the GMs: “It takes me back to those times when you used to come home from the army and you didn’t want to sleep alone. Only with me.”

For the GDs, movement gave them permission to feel, ask, listen: “In the dance there was joy and also longing for each other. It was the first time I allowed myself to miss our being together.” “There was excitement and closeness. There was longing, sadness and a feeling of something having slipped away. Fear, love, thoughts of parting and death arose. All these feelings are familiar. I didn’t allow myself to feel some of them for such a long time.” And, “After the movement I felt more comfortable asking questions about emotional things that don’t normally come up. I wanted to know more about her.”

#### Dance Changes the Older Person’s “State of Mind”

Among most of the GMs, the first meeting with dance gave rise to discussion about physical losses. Complaints such as the following recurred: “I have no feet” and “I have no strength.” Throughout the meeting, however, for all the GMs, their outlook on life was painted in a more positive light. The GDs describe: “The experience is very different, I’m used to hearing stories about difficulties and griping about caregivers and neighbors. This time Grandma chose to tell me about their generous sides.” And, “Grandma remembers dances she danced with Grandpa and talks about happy events in the future. She talks about dances in childhood; I discern a spark in her eyes and excitement, it seems as if the same feeling she once had in her body is present today too.” Most of the GMs shared memories of being courted by the opposite sex and of dances: “Grandma remembers when she danced the tango. She says that Jews used to dance a lot when they came back from Auschwitz. They only wanted to dance. She relates that she danced a lot with her brother who was like a father to her.” And, “As soon as we started to dance, Grandma spoke of the hardship in her life but as the joint movement continued, it surprised me that Grandma started to talk about good things, she recounted how she met Grandpa. This was the first time I heard this story. Then Grandma remembered another story and then another story. It’s amazing how Grandma remembers so many pleasant memories from that time.”

The dance encouraged expression of feelings of love: “In contrast to our regular conversations which are more informational, it surprised me that after the movement, positive feelings and emotional conversation were aroused in Grandma.” “Grandma says that during movement she thought how much she loves me. She can’t stop thanking me for coming. This is a different intimacy than conversational meetings.” And, “In contrast to other times in which Grandma makes gestures that show her love of me, this time she spoke of her positive feelings and her enjoyment of our shared time.” The dance created excitement and emotion, and in a number of cases, the GM wanted to also dance with the grandfather (*N* = 4). For all the GMs, dancing brought greater freedom of movement and physical contact in its wake: “The hug was surprising. The movement facilitated enjoyment, touch and connection. As the meetings continued, the greater use of space was noticeable, as was the longer duration of the movement, larger movements, more variation and changes in movement.” And, “The third time, I feel we are truly dancing together, there is a lot more eye contact and even a bear-hug dance.”

In all cases, the GM felt loved in the dance. Memories of love, nurturing, and concern arose from the GMs: “I was loved at home, I always felt that everyone loves me.” And, the experience of love in the meeting itself: “Grandma says that for her to move together with me makes her feel that she’s not alone, in the movement she thinks of closeness.” And, in her own words: “For me, to dance with my grandchildren and great-grandchildren makes me the happiest in the world. It makes my day and my night.” It was noted that gestures of love were made outside the meeting as well. One GM excitedly related: “Yesterday, at my age, someone asked me to dance.” In almost all of the pairs, the GM wants to brag and proudly tell of the meetings. In a number of cases (*N* = 5), following the meeting with the GD, the grandfather asked to see the GM dance. In one case, the daughter (the mother of the GD) joined the GM in dance classes: “She wanted to do it for a long time already and now it happened.” In a few cases, the meeting aroused curiosity and excitement in the family, and the GM’s continued attempts with dance even after the study ended was made possible by the guidance of the caregiver who lived with the GM.

The joint movement led to uplifted spirits. The GD made statements such as: “In contrast to regular visits, when we dance (and not speak) Grandma feels better, I felt her joy of life, vivacity, smile.” And, “The movement made Grandma happy and excited.” And from the GMs: “Something good happened in my soul, it was good, if it were possible to start the day free-form dancing together with you, it would put me in a good state of mind.” And, “Aside from cooking, I sit all the time. It’s very good to dance, I feel young and it’s even more fun with my granddaughter… to dance with you, (smiles) it’s an experience that certainly must have distorted my features with the smile that I couldn’t stop. (Breathes…) This is a huge gift.” For all of the pairs, dance provided a breath of fresh air. An actual example is brought by one of the GDs: “Grandma smokes several packs of cigarettes a day. It’s like her lifeline. At the end of our meeting, she says that the time passed so quickly and it’s the first time in her life that she completely forgot about her cigarettes.” Movement as food for the soul also emerges from the statement of one of the GDs: “From the moment I enter, I almost always eat something, and in these meetings I didn’t eat anything, maybe the dancing was the food.”

### Unique Elements Contributing to the Meeting’s Success

#### Meeting Framework

All the GMs related to the necessity of their GD’s presence in order for movement to take place. They related to the gap between the longing for a joint dance meeting and the difficulty of moving in an independent manner: “It’s so good, and so hard to do alone.” “I want so much to move freely around the house and don’t do it.” “One quarter hour and it is so meaningful and improves my state of mind. It’s hard to do these things alone.” “If only I were able to dance when you’re not here… with you it’s so enjoyable but I have no patience to do it on my own.” And, “I never dance, only when you’re here.”

The GDs all stressed the need for the regularity of the meetings and their purpose: “I’m excited to come for the third time in a row for a specific purpose, at a regular hour. She opens the door, smiles, is dressed nicely, made up.” And, “The experience for Grandma is a huge thrill. Expectation, pride. She put on makeup, dressed nicely, planned, chose music, called to confirm.” The same things emerged from the GMs: “These are significant days for me. I wait for it. Too bad we can’t continue longer… it’s special.”

In contrast with dance classes, in all the cases, the interaction and pleasure are from dancing and not the performance itself, which is the main point. This is expressed by one of the participating GMs: “I didn’t make any impressive movements or acrobatics… (laughs) I didn’t stand on one leg… but the touch, the movement, the gaze were fun, fun. The hug and the smile were more meaningful.” And, “I went to dance classes but I stopped, it was hard for me. With you it’s extraordinary fun. It worries me that I can’t control my smile. Great pleasure. Celebration. Every grandmother, if they knew, would be jealous of me.”

According to all the participants, the uniqueness of the meeting is in the invitation to free-form dance. As one of the GMs expresses it: “The pleasure derived from the freedom in the movement. You can do whatever you want. Let the body and the music take the lead. I waited for the sessions.” And, “I felt that the movement came from me, to effortlessly do something special. It was spontaneous, unplanned.” Many of the GDs (*N* = 10) emphasized that structured movement (repetition of a series of familiar movements) generally prevented eye contact and touch. Despite the fact that it enabled the GDs and GMs to synchronize their rhythms and forms of movement, it allowed only limited time for an experience of closeness. This contrasts with free-form dance: “I had a feeling of success when at the end of the third dance we did something together, there I felt synchrony, it was pleasant though in the more advanced sessions the need arose for a more spontaneous and close experience. The more spontaneous moments of improvisation create eye contact and closeness.”

#### The Granddaughter’s Instruction

According to all the GDs, their initiation of touch, joint breath, and eye contact are elements they added to the meeting in the goal of encouraging interaction and flow of free-form movement (and not a replay of a regular series of movements). “When I led, eye contact, touch, smile occurred. I see that Grandma also enjoys these and my leading creates a spontaneous dance and brings in other rhythms.” There is an attempt to create eye contact through movement with touch, the GD’s proximity, and the suggestion to use the space of the room. A playful culmination, more grandiose and amusing, made by all the GDs, led to an embrace from their GMs.

All the participants viewed their GM’s leading the movement as the main point of the movement interaction. “Grandma begins to move immediately and to lead the movement which I, with great happiness, join.” When the GD leads, she is attentive to how her GM continues to move in the movement she suggests, so that leading is more of a “suggestion” of movement, which the GM can use when she continues to lead. “I saw that when she borrows movements from me and leads the movement herself, she enjoys it more.” The GDs’ leading included gradual movement, starting with the limbs, then the body, and finally, the pelvis. It was evident that the gap between the generations was free of competition and the need to control: “If this were with my mother it would definitely be uncomfortable but with Grandma, I enjoyed her leading.” The GDs mirror their GM’s movement and suggest slowing it down or making it larger. The changes are small and gradual. The GDs’ movements encourage a sense of stability, crossing movements and rotation, and repetition of the movement on both sides of the body. After each change, the GD makes sure to repeat the movement and create eye contact before another change: “I tried to be attentive to her needs, and at the same time, I attempted to challenge her a little.” The suggestions are characterized by consideration of the GM (“A thought about something flows, an easy and simple movement we can do together.”) and mutuality (“We listened to one another. What felt inappropriate to me, we changed, both of us danced ‘the truth,’ we enjoyed.”). The GDs felt that their presence – showing interest in their GM’s body and her needs – created a space for expressive movement on the GM’s part: “My observance of her, my physical presence together with curiosity about her movement, were experienced as meaningful for my grandmother.”

All the GDs gave their GMs “permission to rest”; it was evident that they paid attention to hints of fatigue and responded to them with a suggestion for an alternative movement – movement while sitting, adding a pillow or a supporting wall: “When Grandma gets tired I stop. Only then does Grandma let go and allow herself to rest.” And, “Despite the fatigue and pain in her knees, Grandma continues a bit more and only when I bring the chair near does she agree to sit down a little.” The GMs all said that this attitude helped them greatly in the movement: “It was very comfortable for me sitting down, it allowed me to relax while I regained strength. I felt that I was still dancing. I lived the rhythm, the tune, the creation. And that’s what enabled me to get back up.” Alongside this, it was seen that all the GDs used words of encouragement and persuasion, highlighting the sense of ability by showing enthusiasm for the body’s movement, such as: “Well done,” “What a beautiful movement,” and “Wonderful, Grandma.”

## Discussion

In the present study, using a qualitative paradigm (“action research”), adult GDs who were dance/movement therapists participated in free-form dance meetings with their GMs. Their professional training notwithstanding, the GDs expressed concern about lacking the tools to lead the meeting towards being a meaningful experience and about being able to safeguard their GM’s health during the course of the meetings. Alongside these concerns, the meetings were found to have significance for the way the GDs perceive aging and their GMs and for the process of eventual parting, an element inseparable from aging. Likewise, the nature of the meetings and the unique instruction (which included regular sessions of free-form dance, joined by the GDs with sensitivity and maintaining their focus on the GM) led to a change in the GMs’ state of mind; it empowered positive outlooks and feelings and expressions of uplifted spirit.

Previous research identified some of the GDs’ concerns; for example, it was found that GCs worry about the health of their GP (Boon et al., 2008) and that they do not possess the skills needed to create an alternative interaction with a GP suffering from dementia (Miron et al., 2019). The present research expands these insights and stresses that concern about successfully creating meaningful interaction is also shared by 30- to 45-year-old GCs. Thus, the invitation to incorporate dance in a meeting aroused worry over the health of the GM, which may be affected due to the physical effort or overwhelming emotion connected with the meeting. Being familiar with these concerns can help in understanding the dynamics of relations that can either prevent or promote closeness and help in creating interventions that support GCs in their support of their GPs.

Specifically, the GDs’ concern that they would not be needed in the meeting and would not be of significance to it (“concern over lack of significance”) should be emphasized. The concern emerged in light of the GMs’ tendency not to make eye contact with them during the dance movements. The disparity between this finding and the finding that discusses the GD’s unique role in the movement meeting (“movement accompanied by a closely related partner”) may find answers in research on physical stability in old age (Rahal et al., 2015). In a study that compared the stability of older people during couple dancing to stability in tai chi practice, it was found that there was greater stability with eyes closed during couple dancing and greater stability with eyes open during tai chi (ibid). It may be hypothesized that the GM’s attempt to imagine the dance steps while maintaining stability is the reason for the frequency of cases in which the GMs did not initiate eye contact while they led the joint dance. Without the knowledge related to old age, this experience is interpreted as rejection and can affect the closeness of the relationship and the degree of satisfaction derived from the joint encounter.

As mentioned, in line with previous studies (Even-Zohar and Tzurit Garby, 2016), the present research indicates that the close relationship between the generations positively contributes to both the GM and the GD. Moreover, the results of the present study reveal the specific advantages of joint dancing for the GD and her GM. For the GD, the dance puts old age in a new light and changes roles and attitudes in the relationship. These changes can lead to closeness even in situations where relations are characterized by emotional distance (Silverstein and Xu, 2016) and endow strength to GCs with respect to fears and anxieties that old age brings up (Miron et al., 2019). The current research also emphasizes the significance of unique experiences for the GM and the GD as part of the GD’s process of leave-taking from her GM and coping with the finiteness of life, and loss – an area that has not, to date, been researched.

Additionally, a previous study highlighted the importance of maintaining close and open discourse between generations (Werner and Lowenstein, 2001), but the understanding of how to promote the features of optimal communication remains open. From the current research, we can learn that joint spontaneous dance not only creates a physical experience but also leads to meaningful interaction that aids in developing discourse and sharing memories. Earlier studies showed that movement arouses emotional discourse (Shuper Engelhard, 2019a) and feelings (Shafir et al., 2016). The present study emphasizes the effect of free-form (in intensity, size, and rhythm of movements), GM-focused dance on awakening memories and positive feelings. Many studies have found that deep breathing and good eye contact are activities that lead to calmness and regulation of the autonomous system and contribute to interpersonal communication (Muro et al., 2016). Furthermore, in research on an older population with dementia, it was found that physical activity reduces the level of depression (De Souto Barreto et al., 2015). The current research reinforces these findings, adding and emphasizing how joint dancing changes the state of mind in old age from repetitive, negative discourse to optimistic, positive discourse.

With respect to the structure of the meetings and the uniqueness of free-form dance as opposed to structured dance, the significance of creativity and playfulness in dance is that they are known to have advantages over synchrony alone, as elements that contribute to the experience of closeness. As extensively described in the findings, the GDs all related to coordination of their movements with their GMs as a pleasant, though insufficient, experience. In many studies, the social advantages of interpersonal synchrony receive broad recognition. In comprehensive literature reviews, it was found that coordinating physical rhythms has a positive effect on relations and on forming empathy (Ramseyer and Tschacher, 2011) and that synchrony contributes to an increase in pro-social behavior, belonging, and group cohesion (Behrends et al., 2012). In addition, there is evidence that synchronous (dance) movement increases endorphin levels and thus serves to relieve pain (Tarr et al., 2015, 2016). Alongside the clear advantages of interpersonal synchrony, a current study found that too much of it may harm the individual’s ability to self-regulate (Galbusera et al., 2019). The present research broadens the understanding of the synchrony component and indicates that without permission to be spontaneous and playful in joint dance, synchrony alone provides a limited experience of shared enjoyment and closeness. Similar findings were obtained in research with couples, which found that the combination of synchrony and permission to spontaneously switch the roles of dance movement leader is a factor in the satisfaction derived from the joint couple experience (Shuper Engelhard, 2018).

It is interesting to note that the research results are based on just three meetings of 10 to 15 min of joint dancing. This expands on and reinforces findings that showed that even very low-level movement leads to an improvement on tests of memory and intelligence in old age (Ratey, 2008), and that short-term intervention with a psychiatric population that included physical activity brought about changes in psychological status (increased level of happiness) and brain activity (EEG showed activity in regions associated with relaxation) (Barton, 2011). Extending this, the present research emphasizes that dancing jointly for a very short amount of time is sufficient for the inter-generational meeting to take on a new cast and for it to bring about a change in the older adults’ state of mind and the GDs’ perception of their GMs.

### Main Conclusion

Joint dancing helps both the GD and the GM improve their quality of life. For the GD, the movement meetings create opportunities for greater closeness with her GM and provide valuable moments in which to work though separation processes, which can be of comfort as the GM’s aging process progresses. For the GM, the dance as a spiritual experience is the “vitality” required for coping emotionally with the losses aging brings. Elements of the dance meeting can be taught by non-experts in dance and movement and thus affect the older person’s interpersonal relationships and quality of life in various and diverse populations. It is important to note that none of the GMs had been a professional dancer, but in her own way, each one was reminded of events connected with dance. This, in practice, is the uniqueness of DMT – it is an emotional encounter through movement that does not require any pre-existing knowledge of dance.

### Limitations, Future Research, and Implications

The present research included only women, all above the age of 30, this with the aim of examining the significance of a free-from dance intervention on the interaction between GDs and their GMs. All the GDs are professionals with expertise who possess the tools, although not defined as such, to conduct a therapeutic-type encounter and to identify therapeutic characteristics during its course. In the future, it is important to examine whether similar findings are obtained with other populations and other age groups and, in doing so, to increase the research population of GCs and GPs.

It can be assumed that extending this research with other, gender-diverse groups will have similar results. These areas are important to research further in order to recognize the unique characteristics of meetings of different types. Despite their professional training, all the participating GDs expressed concern prior to the meetings. It may be assumed that without training in the field, the difficulty of generating a creative and alternative encounter would have been greater. Their training as therapists also likely influenced the manner in which they coped with the emotional content that arose in the meetings. This factor emphasizes the necessity of support circles and sources of knowledge that underpin inter-generational relations between adult GCs and the elderly in the family.

Another significant topic likely to affect results is the use of music. The participants were not instructed in regard to anything excepting the request to be sensitive to their GM’s needs. Mostly, they asked their GM to choose the music or they themselves selected music tied to their GM and her culture. In almost all cases, the GM asked for younger, quicker music than suggested by her GD. It is likely that the memories that came to the fore during the research were influenced by the music, and there is certainly much more to learn about this area through further research.

Likewise, the present study was conducted with a narrow sample and a qualitative paradigm due to the area’s nascence. Follow-up research should employ quantitative analysis to examine the effect of the dance on the GD, the GM, and their relations using physical, cognitive, and emotional measurements and thus expand and build up understanding in the field. Previous studies examined the significance of joint activity of GCs and older people (Majlesi and Ekström, 2016). The choice to focus on free-form dance is based on previous research in the field of DMT in old age (Jiménez et al., 2019). DMT makes an emotional and social contribution in old age and was found to be a factor inhibiting deterioration in regions of the brain affected by dementia (Hole et al., 2015). Positive correlations were also found between movement ability and normal functioning of the temporal lobe, which is related to Alzheimer’s disease (Ratey, 2008). The increase of the proportion of elderly in the population (World Health Organization, 2018), along with the increase in the age group of adult GCs (Kridahl, 2017), necessitates creativity and innovation in providing diverse resources and support. Follow-up studies should examine additional types of joint interventions and compare the effectiveness of different activities.

The current study serves as preliminary research that aims to expand knowledge in the area of gerontology and as the infrastructure for promoting interventions that will support the older person and his or her family. Adopting tools and techniques from the field of DMT to be used in interventions in the community prepares the ground for low-cost support of the elderly with access to many people. Unique DMT techniques include: mirroring and joining the movement; encouraging spontaneous movement; and creativity and attention to emotional content emerging from the movement (Koehne et al., 2016), served by the GDs in dancing with their GMs. Use of movement mirroring is central to the field of DMT. Paying attention to the various articulations of the patient’s body and his or her echo in the therapist’s body contributes to an increase in empathy and self-cohesion (Behrends et al., 2016). All these can support communication between GCs and their GPs.

Beyond the acknowledged contribution of physical activity (Lelièvre et al., 2015), dancing with the GD stimulated positive perception and feelings and created closeness and a spiritual experience – all these can contribute to the older person’s and her family’s quality of life. Familiarity with the concerns of the generation of adult GCs can help build support frameworks adapted to their needs and thus reduce avoidance of creating meaningful relations and engaging in joint activities with the GP. The unique framework of the meeting (a regular structure that combines free-form dance with a closely related person who mirrors the GM’s movements, encourages playfulness and touch, gives permission to rest and bolsters abilities) promoted physical activity even when the body was fatigued and weak and in circumstances where the GM was not accustomed to participating in other physical activities. This emphasizes the significance of the close and familiar relationship as a means to promote new experiences (which can occasionally seem impossible) for the older person. The research indicates the need to recruit the generation of GCs to fortify the spirit, creativity, and optimism in old age, and the necessity of developing models of community intervention to provide support for the GCs’ generation. This should include space for working out concerns and learning unique techniques and communication alternatives in old age.

## Data Availability Statement

The datasets generated for this study are available on request to the corresponding author.

## Ethics Statement

The studies involving human participants were reviewed and approved by the Kibbutzim College of Education. The patients/participants provided their written informed consent to participate in this study.

## Author Contributions

ES initiated and designed the study.

## Conflict of Interest

The authors declare that the research was conducted in the absence of any commercial or financial relationships that could be construed as a potential conflict of interest.
